# Clinical Overlaps in Reticulate Pigmentary Disorders: A Study of Three Cases

**DOI:** 10.7759/cureus.56600

**Published:** 2024-03-20

**Authors:** Nishtha Malik, Rahul S Nair, Aravind Reddy, Pooja Chaurasia, Niranjana S Pillai

**Affiliations:** 1 Dermatology, Venereology, and Leprosy, Dr. D. Y. Patil Medical College, Hospital & Research Centre, Dr. D. Y. Patil Vidyapeeth, Pune (Deemed to be University), Pune, IND; 2 Orthopedic Surgery, Holy Cross Hospital, Kollam, IND

**Keywords:** dowling-degos disease, reticulate acropigmentation of dohi, reticulate pigmentary disorders, dyschromatosis symmetrica hereditaria, reticulate acropigmentation of kitamura

## Abstract

Reticulate pigmentary disorders are autosomal dominant pigmentary disorders caused by abnormalities in the keratin 5 and keratin 14 genes. Here, we report three cases of reticulate hyperpigmentation disorders with clinical overlaps of the reticulate acropigmentation of Kitamura, Dowling-Degos disease (DDD), and dyschromatosis symmetrica hereditaria (DSH), all three having limited treatment options.

## Introduction

Reticulate pigmentary disorders refer to a group of pigmentary disorders that are inherited in an autosomal dominant manner. The conditions encompass Dowling-Degos disease (DDD), the reticulate acropigmentation of Kitamura, the reticulate acropigmentation of Dohi, Galli-Galli disease, and Haber's syndrome [1].

The majority of prevalent hereditary reticulate pigmentary illnesses exhibit abnormalities in the keratin 5 and keratin 14 genes. A mutation in the keratin 14 gene leads to a reduced amount of functional protein, making the keratinocyte more susceptible to stimuli that promote cell death. On the other hand, a mutation in the keratin 5 gene increases the likelihood of undergoing changes in the epithelial structure, as well as causes the misplacement of melanin-containing organelles and plays a crucial part in their transportation. This indicates a flaw in the interaction between keratinocytes and melanocytes, which might subsequently impact the regulation of pigment in melanocytes. Recently, we happened to see three cases of reticulate hyperpigmentation with clinical overlaps [2].

## Case presentation

Case 1

**Figure 1:**
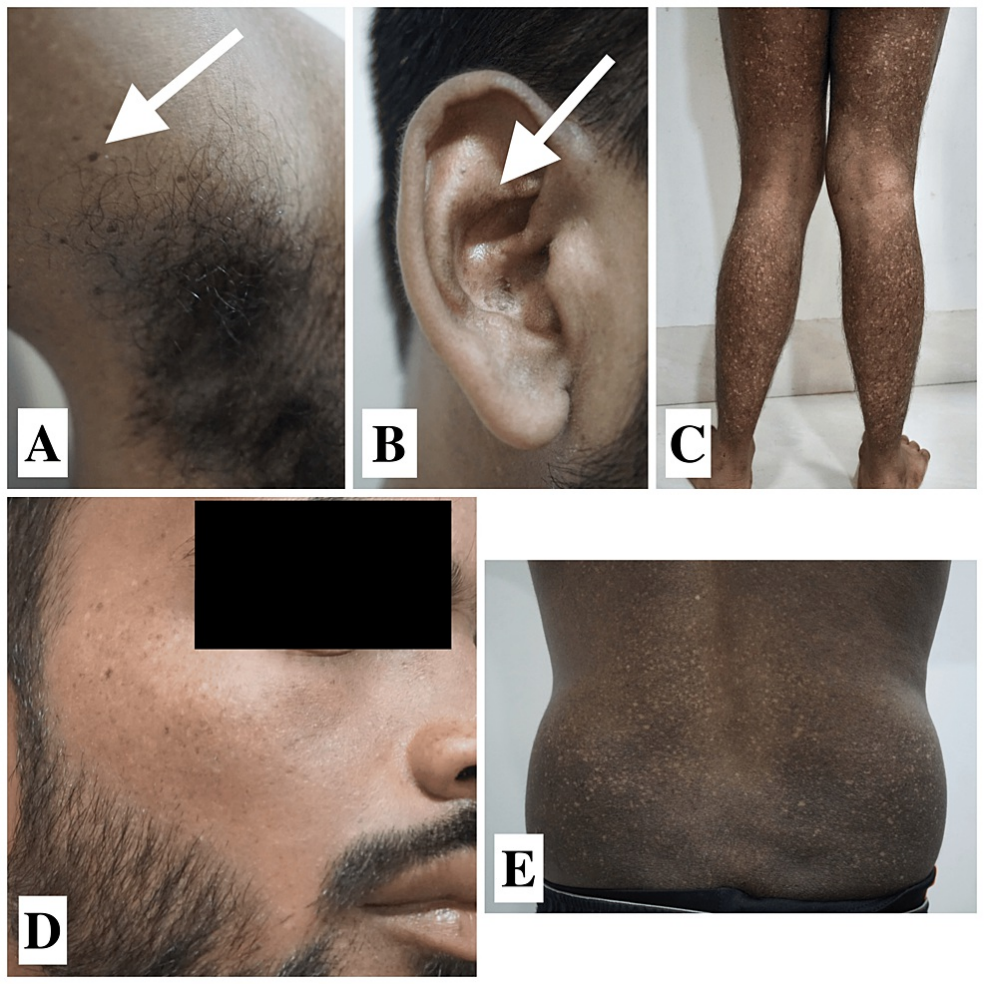
Clinical images of case 1 (A) The white arrow showing keratotic papules in the axilla. (B) The white arrow showing multiple well-defined hyperpigmented macules with comedo-like lesions in the right ear. (C) Multiple well-defined hyperpigmented, hypopigmented, and depigmented macules over the bilateral lower limbs. (D) Multiple well-defined hyperpigmented macules over the right malar area. (E) Multiple well-defined hyperpigmented, hypopigmented, and depigmented macules over the back

**Figure 2:**
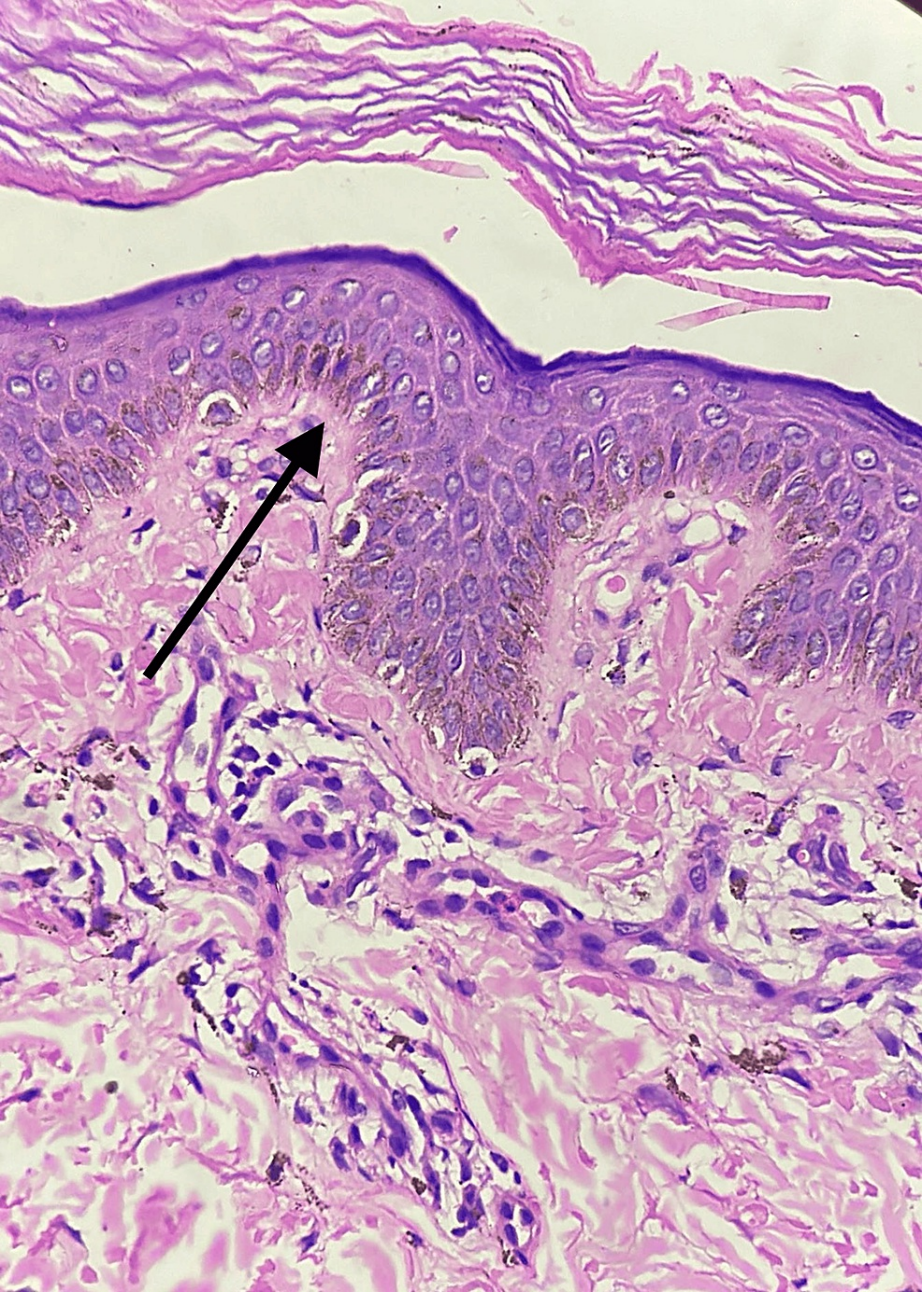
Histopathological image of case 1 The black arrow showing hyperkeratosis and hyperpigmentation of basilar keratinocytes in the epidermis, mild melanin incontinence, and perivascular chronic inflammatory infiltrate in the dermis (H&E: ×400) H&E: hematoxylin and eosin

A 22-year-old young male presented with dark- and light-colored skin lesions throughout his body since adolescence. The lesions first manifested when he was 12 years old, appearing on the dorsal aspect of his hands and feet. Over time, the lesions have spread from the distal areas to the proximal areas, affecting the entire body and the cheeks. The patient was a product of a non-consanguineous marriage and has no family history of such lesions. On examination, multiple well-defined hyperpigmented and depigmented macules were found over the malar area of the face, abdomen, back, and bilateral upper and lower limbs with keratotic papules over the bilateral axillae and comedo-like lesions inside the ears (Figure 1). Histopathological examination reveals mild hyperkeratosis and mild hyperpigmentation of basilar keratinocytes in the epidermis, mild melanin incontinence, and mild perivascular chronic inflammatory infiltrate in the superficial dermis (Figure 2).

Case 2

**Figure 3:**
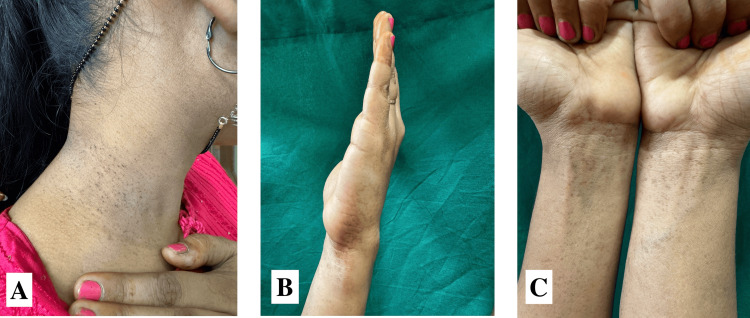
Clinical images of case 2 (A) Black comedo-like lesions around the neck. (B) Multiple well-defined hyperpigmented macules over the medial aspect of the dorsum of the left hand. (C) Multiple well-defined hyperpigmented macules over the flexure aspect of the distal forearms

**Figure 4:**
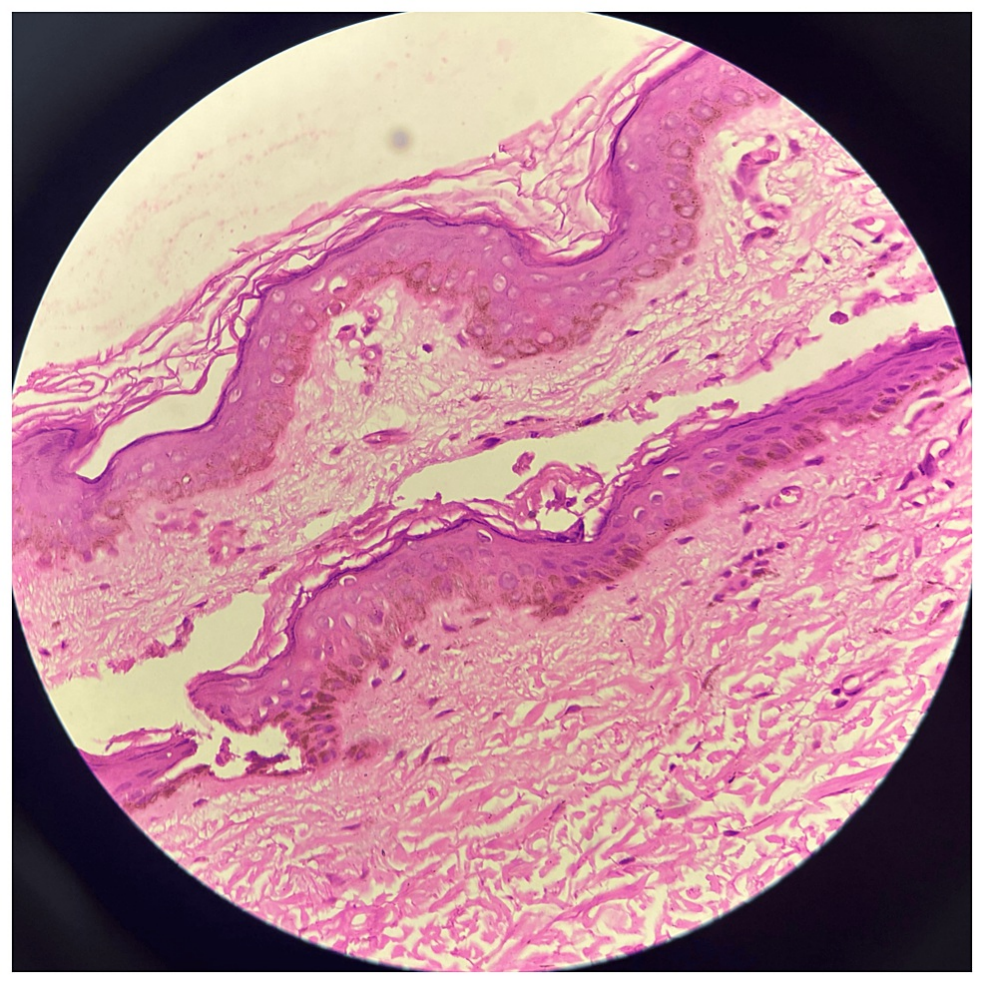
Histopathological image of case 2 Epidermal flattening and loss of rete ridges with mild atrophy of the lining epithelium and mild vascular proliferation of the papillary dermis (H&E: ×400) H&E: hematoxylin and eosin

A female, 18 years of age and from a non-consanguineous marriage, presented with dark-colored lesions on her neck and forearm that have been present for a duration of two years and aggravated in summers. Upon inquiry, there is no family history of such lesions. On examination, multiple well-defined hyperpigmented macules were present over the flexure aspect of the distal forearms and the medial aspect of the dorsum of the hands, along with black comedo-like lesions around the neck (Figure 3). On histopathology, the epidermis shows flattening and loss of rete ridges with mild atrophy of the lining epithelium and mild vascular proliferation in the papillary dermis, and the dermis shows hair follicles and follicular plugging (Figure 4).

Case 3

**Figure 5:**
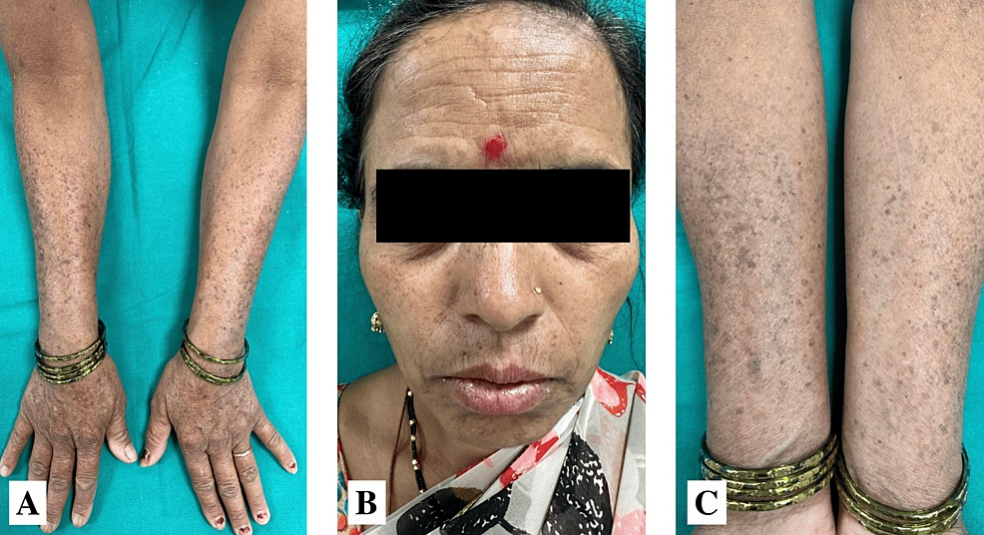
Clinical images of case 3 (A and C) Multiple well-defined hyperpigmented macules over the bilateral forearm and dorsum of the hands. (B) Multiple well-defined hyperpigmented macules over the face

**Figure 6:**
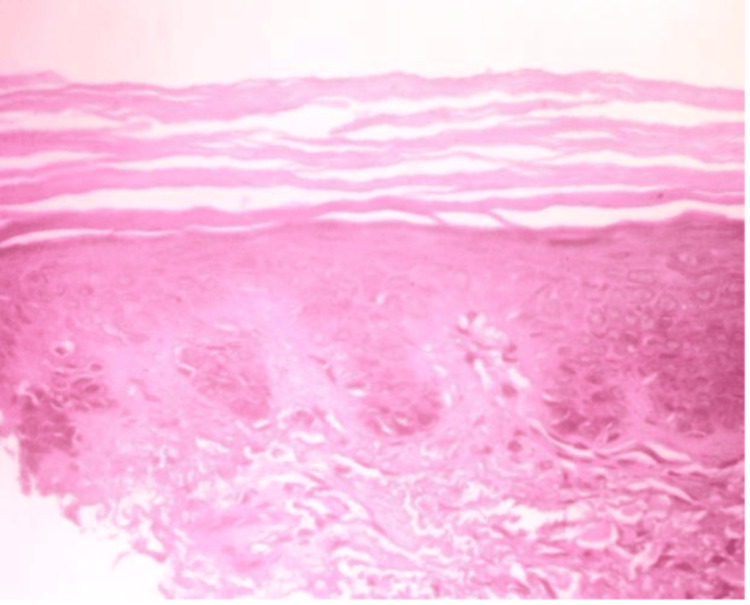
Histopathological image of case 3 Hyperkeratosis and mild hyperplasia of the epidermis, along with elongated, bulbous rete ridges with significant pigmentation at the tips. The dermis shows minimum superficial perivascular lymphocytic infiltrate (H&E: ×400) H&E: hematoxylin and eosin

A 38-year-old female came to us with dark-colored lesions on her forearms and face over a period of two months. She was born from a non-consanguineous marriage, and her father had similar lesions. On, examination, multiple well-defined hyperpigmented macules were seen over the bilateral forearm, dorsum of the hands, and face (Figure 5). Histopathological analysis showed hyperkeratosis and mild hyperplasia of the epidermis. However, horn cysts were not seen. The rete ridges were elongated and bulbus with marked pigmentation at the tips. The dermis has minimum superficial perivascular lymphocytic infiltrate; however, pigment incontinence or melanophages were not observed (Figure 6).

## Discussion

Kitamura and Akamatsu documented the initial account of the reticulate acropigmentation of Kitamura in Japan in 1943. It usually appears throughout the first and second decades of life. The dorsum of the hands and feet exhibit a network of pigmentation resembling freckles, along with palmoplantar pits and disrupted dermatoglyphics. The pigmented skin abnormalities progressively intensify over time, and exposure to sunlight might exacerbate the condition. Histopathological studies reveal basal layer melanocyte clusters and epidermal atrophy together with the clublike extension of rete ridges [1].

Dowling initially coined the term "Dowling-Degos disease" in 1938 and subsequently introduced the term "Degos" in 1954. The condition is characterized by the presence of reticular hyperpigmentation in the flexor regions, specifically the neck, axilla, antecubital fossa, inframammary areas, and groin. The face and neck may exhibit black comedo-like lesions, along with pitted perioral acneiform scars. The onset might occur anywhere from adolescence to late age [1]. On the histological analysis, DDD is marked by a basal layer pigmentation increase, rete ridge downward elongation, and suprapapillary epithelium thinning. This typically emerges from the dermis and hair follicles looking like antlers or filiform structures. In addition, there is mild perivascular lymphohistiocytic infiltration and dermal melanophages [3].

Dyschromatosis symmetrica hereditaria (DSH), also referred to as the reticulated acropigmentation of Dohi, is an inherited condition that follows an autosomal dominant pattern, manifesting with small hypo- and hyperpigmented macules, with uneven size and shape, symmetrically scattered on the dorsum of the hands and feet. Lesions occur in childhood, typically before the age of six, and persist throughout life without any alterations in color or distribution after reaching stability in adolescence [4]. The histology of the lesions reveals either elevated or reduced basilar pigmentation in the hyperpigmented or hypopigmented lesions, respectively.

There is a convergence of these illnesses in all three patients as summarized below (Table 1).

**Table 1:** Epidemiological, clinical, and diagnostic summary of the three cases

	Case 1	Case 2	Case 3
Age (years)/sex	22/male	18/female	38/female
Onset	Puberty	Two years	Two months
Family history	Absent	Absent	Present (in father)
Sites	The malar area of the face, both ears, abdomen, back, axillae, and both upper and lower limbs	Neck, flexure aspect of the distal forearms, and medial aspect of the dorsum of the hands	Bilateral forearm, dorsum of the hands, and face
Features of the lesions	Hyperpigmented, hypopigmented, and depigmented macules; keratotic papules; comedo-like lesions	Hyperpigmented macules and black comedo-like lesions	Hyperpigmented macules
Histopathology	Mild epidermal basilar keratinocyte hyperkeratosis and hyperpigmentation, modest melanin incontinence, and superficial dermis perivascular chronic inflammatory infiltration	Epidermal flattening and loss of rete ridges with slight atrophy of the lining epithelium; mild vascular proliferation in the papillary dermis; dermal hair follicles and follicular plugging	Epidermal hyperkeratosis and modest hyperplasia; long, bulbous rete ridges with significant pigmentation at the tips; minimum superficial perivascular lymphocytic infiltration in the dermis
Histopathological findings	Dyschromatosis symmetrica hereditaria	Dowling-Degos disease	Dowling-Degos disease
Clinical overlaps	Dyschromatosis symmetrica hereditarian: the presence of hypopigmented lesions; Dowling-Degos disease: keratotic papules on axillae and comedo-like lesions inside the ears	Dowling-Degos disease: black comedo-like lesions on the neck; the reticulate acropigmentation of Kitamura: freckle-like lesions on the dorsum of the hands and aggravation in summer	The reticulate acropigmentation of Kitamura: only freckle-like lesions present over the forearms, hands, and face

All sorts of therapies are inefficacious. Several topical treatments, including azelaic acid, retinoic acid, hydroquinone, corticosteroids, and systemic retinoids, have been used but have not shown considerable effectiveness. However, there is just one recorded case of using an erbium-doped yttrium aluminum garnet (Er:YAG) laser with pulse energies of 1,000 and 1,200 MJ. The specific therapy produced positive outcomes after three successive iterations. Using fractional lasers can help improve the response in the atrophic lesions [2].

## Conclusions

Though reticulate pigmentary disorders refer to a group of distinct pigmentary disorders, more often than not, they clinically present as overlaps. Such overlaps of features have uncommonly been previously reported in the literature.

Even though there is no substantial mortality linked with these conditions, it can cause great anguish and worry in patients; therefore, while doing examinations, dermatologists must be wary of such presentations in order to make an early diagnosis and initiate multidisciplinary therapy to alleviate patient distress.
